# Quantifying time spent outdoors: A versatile method using any type of global positioning system (GPS) and accelerometer devices

**DOI:** 10.1371/journal.pone.0299943

**Published:** 2024-05-03

**Authors:** Wei Liu, Timothy Chambers, Kimberly A. Clevenger, Karin A. Pfeiffer, Zachary Rzotkiewicz, Hyunseo Park, Amber L. Pearson

**Affiliations:** 1 China Institute of Water Resources and Hydropower Research, Beijing, China; 2 Department of Geography, Environment & Spatial Sciences, Michigan State University, East Lansing, MI, United States of America; 3 Department of Public Health, University of Otago, Wellington, New Zealand; 4 Department of Kinesiology and Health Sciences, Utah State University, Logan, UT, United States of America; 5 Department of Kinesiology, Michigan State University, East Lansing, MI, United States of America; 6 Harman International, Stamford, Connecticut, United States of America; 7 CS Mott Department of Public Health, Michigan State University, Flint, MI, United States of America; Universiti Malaysia Terengganu, MALAYSIA

## Abstract

Spending time outdoors is associated with increased time spent in physical activity, lower chronic disease risk, and wellbeing. Many studies rely on self-reported measures, which are prone to recall bias. Other methods rely on features and functions only available in some GPS devices. Thus, a reliable and versatile method to objectively quantify time spent outdoors is needed. This study sought to develop a versatile method to classify indoor and outdoor (I/O) GPS data that can be widely applied using most types of GPS and accelerometer devices. To develop and test the method, five university students wore an accelerometer (ActiGraph wGT3X-BT) and a GPS device (Canmore GT-730FL-S) on an elastic belt at the right hip for two hours in June 2022 and logged their activity mode, setting, and start time via activity diaries. GPS trackers were set to collect data every 5 seconds. A rule-based point cluster-based method was developed to identify indoor, outdoor, and in-vehicle time. Point clusters were detected using an application called GPSAS_Destinations and classification were done in R using accelerometer lux, building footprint, and park location data. Classification results were compared with the submitted activity diaries for validation. A total of 7,006 points for all participants were used for I/O classification analyses. The overall I/O GPS classification accuracy rate was 89.58% (Kappa = 0.78), indicating good classification accuracy. This method provides reliable I/O clarification results and can be widely applied using most types of GPS and accelerometer devices.

## Background

Increasingly researchers are interested in quantifying time spent outdoors as a metric of contact with nature. Studies suggest that spending time outdoors is associated with increased time spent being physically active and with lower depressive symptoms and chronic disease risk [1, 2]. In fact, spending 120 minutes outdoors per week is associated with improved health and wellbeing [3]. Such findings could prove useful in setting recommendations for time spent outdoors, similar to recommendations for physical activity (PA). However, many studies rely on self-reported measures of time spent outdoors, which is prone to recall bias and may impact the epidemiological findings associating time outdoors with positive health outcomes.

To address potential recall bias, some studies have used global positioning system (GPS) data to quantify time spent outdoors. GPS data help us understand human behavior by providing both time and location information of high accuracy. Studies using GPS data have used algorithms relying on the GPS device’s Signal to Noise Ratio (SNR), which is a measure of the magnitude of the signal received by the GPS tracker, as one of the key variables to classify time spent in indoor or outdoor environments [4, 5]. The Personal Activity Location Measurement System (PALMS) algorithm also uses SNR to differentiate indoor and outdoor points [6–10]. Lam, et al. [11] compared time spent outdoors through PALMS using a 250 SNR threshold and the in-vehicle trip detection against images captured from automated cameras, which resulted in 80.9% overall accuracy. However, not all GPS devices provide satellite-signal-related data, which has limited SNR-based algorithm’s widespread applicability. Although using SNR is optional in PALMS and one study found that PALMS performed similarly well without using SNR [6], disadvantages of PALMS include that it is a server-based system, with the previous UCSD web-application no longer available, and setting up the PALMS on a researcher’s PC requires certain technical skills. Also, PALMS aggregates data into 1-minute level, which is less flexible if higher temporal resolution is required.

Studies investigating time spent outdoors often have a dual interest in outdoor physical activity thus, they use both GPS data and accelerometer data linked by timestamps. Many accelerometers also add the benefit of containing a light sensor which measures the intensity of light in lux (lx) units that can also be used for outdoor data classification [12, 13], although there is no consensus on the threshold of lux to distinguish outdoor data classification [12]. Challenges with the usage of lux data include that the sensor can be covered by clothing, so some studies collected data under controlled experiment environments to ensure the sensor was exposed [7, 13], or excluded low-temperature days to reduce the chance of clothing covering it when collecting participant data in the field [12]. A study of preschoolers compared GPS data classified as occurring outdoors by using SNR versus lux data and found that both methods had moderate to high levels of accuracy [7], but the data from both devices were not combined and used together to test if classification accuracy can be further improved.

The current study aimed to develop a novel method to classify time spent outdoors (non-car time) using linked GPS and accelerometer data, not relying on SNR or the PALMS algorithm. Such a method can be widely applied to field data, regardless of brand of GPS device or accelerometer and without reliance on participant recall (travel diaries). To achieve this, we conducted a controlled experiment among university student volunteers to test the validity of the method developed, which incorporated cluster detection, removal of car travel, and spatial overlay analyses. We then validated the method using semi-real-time self-reported travel diary data, with the intention that future studies would not require collection of such data.

## Methods

### Data collection and preparation

Five adult university students wore an accelerometer (Actigraph wGT3X-BT) and a GPS device (Canmore GT-730FL-S) on an elastic belt over their right hip for two hours in June 2022. The participants logged their activity mode, setting, and start time via a travel diary every time they changed activity mode. The research team scheduled a time with each participant and asked the participant to finish the experiment within 24 hours. Participants were instructed to wear the belt over their clothes for two consecutive hours and do the following activities at least once for a minimum of five minutes in the order of their choice with no time gap between two activities: 1) engaged in physical activity (e.g. walking) in an indoor environment; 2) engaged in physical activity (e.g. walking) in an outdoor environment; 3) driving on a major road; 4) staying sedentary in an indoor environment; and 5) staying sedentary in an outdoor environment. Before switching to a different activity, participants would take a screen capture of the local time (https://time.is) using their mobile phone, which helped them accurately log their activity diaries to a mobile application called ArcGIS Survey123 (version 3.16.114, Copyright © 2022 Esri Inc) after the two-hour period ended. Information submitted to Survey123 included the experiment start time, start time of each activity and screen captures, activity mode, and time when experiment ended and belt was removed. All data collections were done within East Lansing, Michigan, USA. This experiment was approved by MSU IRB (STUDY00007551) and each participant signed a consent form.

Accelerometers were initialized to collect data 30 times per second (30 Hz). Data were downloaded and re-integrated as activity counts in 5-second epochs using ActiLife software (version 6.13.4). GPS devices were initialized to collect data every 5 seconds. Timestamps in the raw GPS data were rounded to the nearest 5 seconds to be linked to accelerometer data (called linked-data hereafter). These GPS data underwent noise removal and imputation (see S1 File) prior to linkage with accelerometer data.

### Destination detection

**Fig 1:**
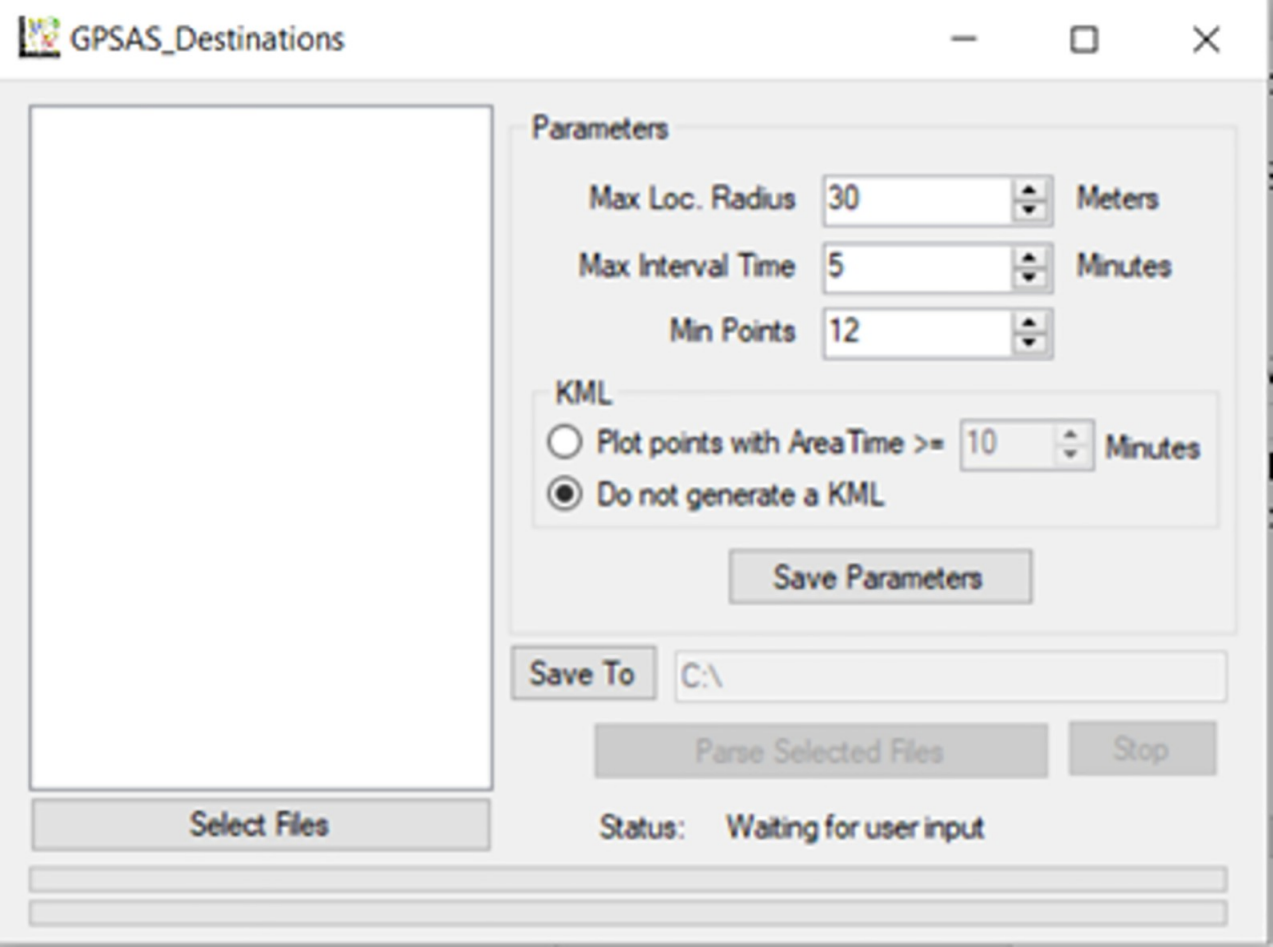
GPS classification rules (dash-line boundaries indicate skippable steps if building footprint data are not available).

**Fig 2:**
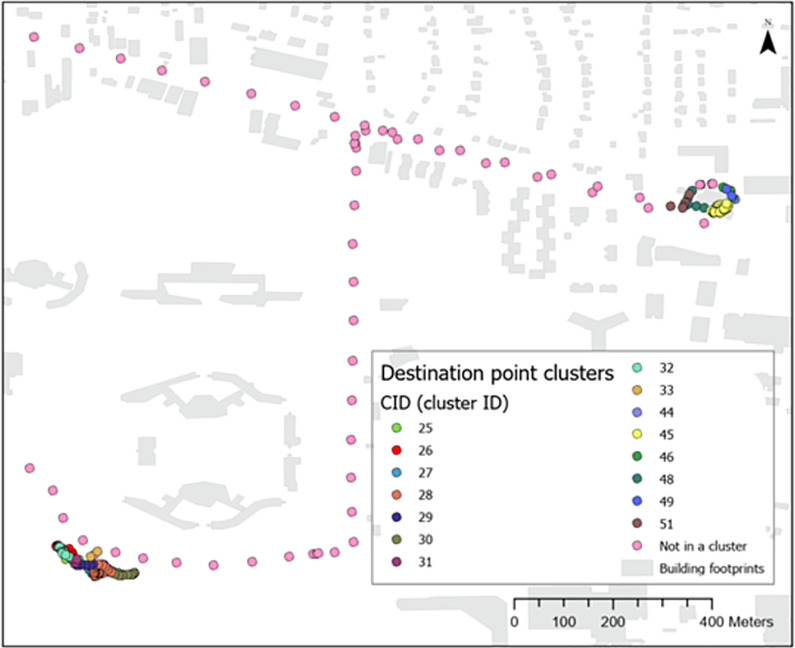
GPSAS_Destinations interface.

Next, a rule-based point cluster-based method was developed to identify indoor, outdoor, and in-vehicle time (Fig 1). The first step was to identify locations visited, as most destinations are indoor locations [14]. We used an open-source Virtual C# application called GPSAS_Destinations Version 1.8.1 (https://github.com/zackrzot/GPSAS_Destinations) to identify space-time clusters of GPS data. This application was developed based on Spatial-Temporal Density-Based Spatial Clustering of Applications with Noise (ST-DBSCAN) algorithm to group points into clusters based on provided space and time constrains with each cluster labeled as a destination. In our analysis, we set a minimum number of 12 GPS points (equal to 1 minute for 5-second-interval GPS data) within a 30-meter-radius area and with a maximum of 5-minute interval time. This parameter combination was selected based on our pilot tests using datasets collected by our researchers. Fig 2 shows the interface of the GPSAS_Destinations application.

The space-time clustering analysis generated four outcome variables as explained below, which were used in designing the I/O classification rules. (1) AID: a unique area ID that identifies a unique destination a single cluster of GPS points was placed in. (2) CID: a unique cluster ID that identifies a single cluster of GPS points that satisfy the space-time parameters. (3) InstanceTime: The amount of time spent at a cluster (CID) in minutes. (4) AreaTime: The cumulative time spent at this destination (AID) across clusters in minutes.

### Cluster-based I/O classification

**Fig 3:**
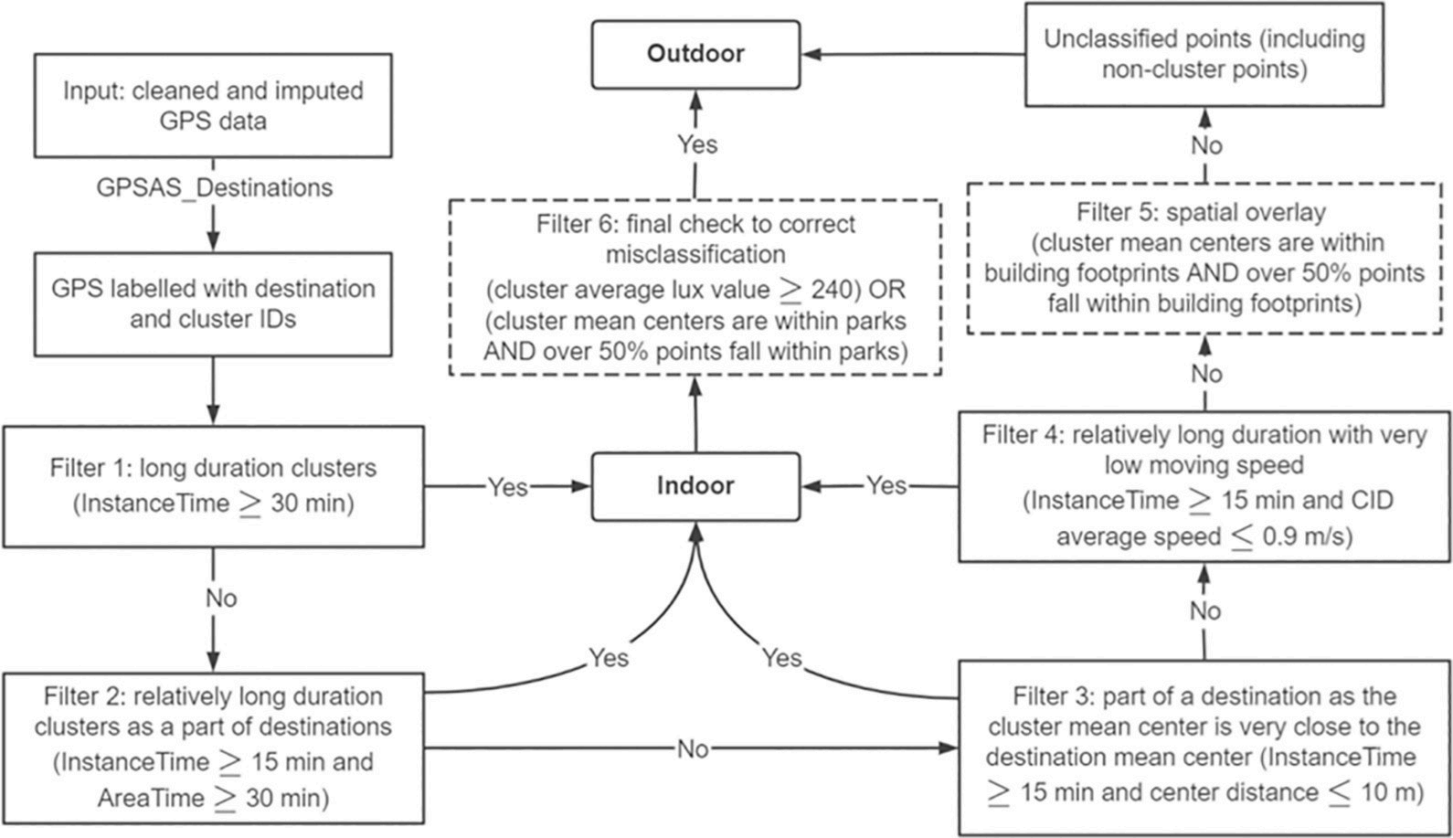
Examples of point clusters identified by GPSAS_Destinations.

Based on the space-time clustering results, the I/O classification steps are shown in Fig 3. Filter 1 identifies clusters lasting for a long period of time (30 minutes). Filter 2 identified clusters of a moderate period (over 15 minutes) but also was part of a major destination (area time over 30 minutes). Filter 1 and 2 clusters were classified as indoor points under the assumption that it is unlikely a participant in an outdoor environment would remain stationary within a 30-meter circular area for a long time. Filter 3 calculated the distance between the spatial mean centers of a cluster and its corresponding destination. Clusters that are close to their associated destination (< = 10 meter) and lasted for 15 minutes or longer were treated as an indoor cluster. For activities such as shopping in a grocery store, where a destination did not consist of many clusters (i.e. AreaTime was not much longer than InstanceTime), filter 4 was able to distinguish those indoor points by specifying a relatively long cluster time (> = 15 minutes) and a low average speed (0.9 m/s which is a reference walking speed of senior adults [15, 16]. Lastly, to distinguish other indoor GPS points that were not picked out by previous filters, we applied another spatial check by overlaying the GPS points with the East Lansing building footprint data, and a cluster was marked as indoor if the cluster mean center as well as over 50% of points from this cluster fell within a building footprint. After the fifth filter, all the non-indoor clusters and points that were not identified as destination/cluster points were set as outdoor data. Given indoor clusters could be misclassified, two additional filters were applied to correct errors. First, indoor clusters with an average lux value over 240 (Flynn et al., 2014) were reclassified as outdoor data. Second, clusters located within a park (cluster mean centers and over 50% points of the cluster falling within park) were reclassified.

### In-vehicle time

To identify in-vehicle time (which is typically omitted from outdoor time research), we aggregated GPS from 5-second data to 1-minute data and calculated average speed within the minute. If the average speed was over 6.944 m/s (25 km/h), all GPS points within the minute were classified as driving mode. The threshold of 6.944 m/s was determined based on the default car time speed threshold used in Personal Activity Location Measurement System (PALMS) version R4 (Kang et al., 2018). To address scenarios such as temporary slow-down period such as encountering a traffic light, we used the following rule to further classify the GPS data: if a non-driving bout of GPS points lasted less than 3 minutes, and was directly bounded by two driving bouts with either block lasting over 2 minutes, the non-driving bout was then considered as a part of the driving trip and classified as car time.

### Validation

**Fig 4:**
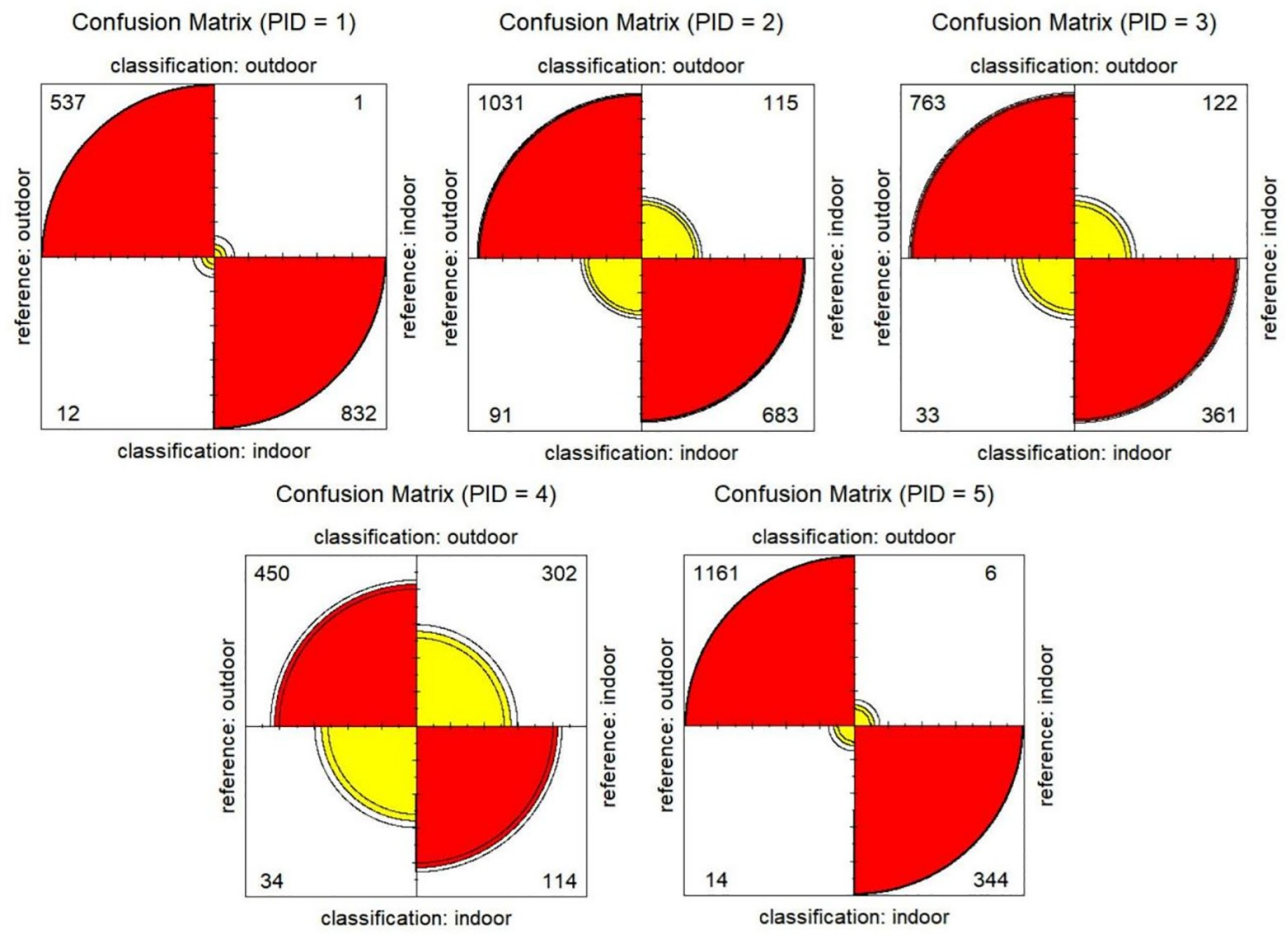
Confusion matrices (numbers in the figure are counts of GPS points per category. Red: correctly classified; Yellow: misclassified).

The activity diaries submitted via Survey123 were exported and the timestamps when each activity started were also rounded to nearest 5 seconds so that the data can be formatted as 5-second-interval data to link to GPS and accelerometer data for validation. Confusion matrices were constructed for each of the participants and all participants’ data were combined to calculate classification accuracy as well (Fig 4).

### Software

GPS outlier labeling, imputation, linking with accelerometer data, rule-based I/O classification and validation were done using R. Manual noise labeling was done in ArcGIS Pro 2.7. Space-time clustering was processed in GPSAS_Destinations 1.8.1.

## Results

Five participants collected a total of 6,911 GPS points, equal to 575.92 minutes, and averaged as 1,382 points (115.18 minutes) per person. After noise removal (n = 1 points) and GPS imputation (n = 96 points), a total of 7,006 points for all participants were used as the input for GPSAS_destinations and I/O classification analyses. There were no missing data for the required information in the activity dairy and all self-reported timestamps were consistent with the submitted screen captures of local time.

As shown in Table 1, the overall I/O GPS classification accuracy rate is 89.58% (Kappa = 0.78), indicating good classification accuracy [17]. Specifically, the user’s accuracy (i.e., the probability that a value predicted to be in a certain class really is that class) of outdoor points (87.83%) is lower than the producer’s accuracy (i.e., the probability that a value in a given class was classified correctly) (95.54%). The overall accuracy among most participants, ranged from 87.9 to 99.06%, except one participant (ID = 4, overall accuracy = 62.67%, Kappa = 0.21).

**Table 1:** Summary of indoor/outdoor GPS classification accuracy rates.

Participant ID	User’s accuracy (%)		Producer’s accuracy (%)		Overall accuracy (%)	Kappa
	Outdoor GPS	Indoor GPS	Outdoor GPS	Indoor GPS		
1	99.81	98.57	97.81	99.88	99.06	0.98
2	89.97	88.24	91.89	85.59	89.27	0.77
3	86.21	91.62	95.85	74.74	87.88	0.73
4	59.84	77.03	92.98	27.4	62.67	0.21
5	99.49	96.09	98.81	98.29	98.69	0.96
All combined	87.83	92.69	95.54	81.04	89.58	0.78

## Discussion

The I/O classification results showed high accuracy rate in general. After plotting the GPS points and check against the map and satellite imagery, the followings are major reasons that contribute to the errors: (1) some reported indoor GPS points drifted randomly outside of the building with high speed probably due to signals being blocked by the building; (2) some reported indoor points were misclassified as outdoor points due to high average lux value, which might result from some points in the cluster have very high lux value due to being close to the entrance or some floor-to-ceiling windows, or due to the bright fluorescent lights such as in a grocery store; or (3) the participant accidentally picked the wrong mode from the drop-down list when filling the survey. The first two are the main reasons why the classification accuracy rate of participant 4 was much lower than others. We did a sensitivity comparison test to remove optional filters. When filter 5 was removed, which means building footprints were not used to check point clusters, the overall accuracy was 83.31% and Kappa value was 0.64. When we removed the lux-based reclassification step in filter 6, the overall accuracy and Kappa value dropped from 89.58% and 0.78 to 83.80% and 0.67 respectively. When the park overlay analysis was removed from filter 6, the overall accuracy and Kappa value did not change. The above analyses indicate that even though misclassification could occur, using the lux value and building footprint data in I/O classification increased classification accuracy overall and doing spatial overlay for parks could be omitted for our example dataset.

The spatial overlay analyses using building and park footprint data was a latter step and used as optional approach for the following reasons. Firstly, not all research projects can access building and park footprint data for their study area. This step can be skipped if data are not available, and the majority of clusters can still be distinguished using previous filters. Secondly, the footprint data is often a secondary data source which may be outdated, leading to misclassification. Thirdly, in some scenarios, such as within an apartment building where the GPS accuracy is low, the displacement of GPS points can cause indoor points/clusters being far from the building and won’t be identified as indoor by solely using this spatial overlaying filter.

In general, the cluster-based GPS classification method provided good I/O classification results for our testing data, and this method has some strengths. The GPSAS_Destinations and R are both open-source applications, which automated the process and completed the classification in a short amount of time at a minimal cost. For our dataset (7,006 points), the GPSAS_Destinations took 73 seconds and R code took 5 seconds to run respectively. Unlike point-based classification, each activity mode in the classified outcome consists of points with continuous timestamps (lasting at least 1 minute). The method also offers flexibility of using customized parameters and cutoff values to suit projects from different study areas (places with beaches or congested traffic) or on different participant groups (such as children or senior adults). For studies using GPS trackers at a lower cost but no SNR data, this approach provides an alternative to effectively classify I/O GPS data.

We acknowledge that this study has some limitations. Rules and thresholds in the method can largely affect the classification accuracy. Depending on the study area and lifestyle or behavior patterns of participants, modification of rules and thresholds are necessary, although the framework for building filtering rules based on destination clusters can be easily applied to other study areas. Using the lux value in the classification is optional but our analysis showed that the lux data from ActiGraph accelerometers can improve the classification accuracy by 5.8%. For studies using accelerometers from other brands or models without light sensors, the proposed method can still by applied but the overall accuracy might be affected. Using building footprint data can improve the overall accuracy by 6.27% for our dataset. Building footprints can be extracted from remote sensing images and there are open-source building footprints datasets available to use directly (e.g. Microsoft maps released country-wide open building footprints datasets in the US, see https://github.com/microsoft/USBuildingFootprints). Although around 7000 points were used for testing, data were only collected from five participants with certain activities conducted within two hours as required by the experiment design. A future study based on data in the field and activity diaries of a larger sample size is helpful to further improve the method.

Despite these limitations, using the GPSAS_Destinations to identify point clusters is the key step of this method, and the identified destination cluster information as the intermediate data and point attributes can be useful for research that requires designation of indoor and outdoor GPS data. Secondly, this method can be applied using most types of GPS and accelerometer devices. Also, filters and rules are flexible and customizable so users can extend or modify the framework according to their prior knowledge about their study site(s) and their particular participants’ travel and activity patterns.

## Conclusion

In this study we proposed a point-cluster and rule-based method to classify I/O GPS data using linked GPS and accelerometer data from participants outside of controlled experiments. According to our validation analysis, the method provides reliable I/O clarification results (overall accuracy = 89.6%) and can be widely applied using most types of GPS and accelerometer devices.

## Supporting information

S1 Checklist STROBE statement—checklist of items that should be included in reports of observational studies. (DOCX)

S1 File GPS noise removal and imputation rules. (DOCX)
