# Supplementary material for: Quantifying time spent outdoors: A versatile method using any type of global positioning system (GPS) and accelerometer devices
Source: PLoS One. 2024 May 3;19(5):e0299943. doi: 10.1371/journal.pone.0299943 (PMC11068186; doi:10.1371/journal.pone.0299943)
Supplement: S1 File — (DOCX) [file pone.0299943.s002.docx]

Supplementary Materials

1. GPS noise removal
   1. Labeling outliers using moving window

Firstly, we calculated time interval, distance between two consecutive GPS points, and speed (distance/time interval) for the raw GPS data. A sliding window method was used to label outliers with a window size of 10 records.

The outlier detection used an interquartile range (IQR) criterion, which is the rule used in box plots. Specifically, for a subset of n observation (Zi and its n-1 predecessors), an outlier has a value > Q0.75+1.5*IQR or < Q0.25−1.5*IQR (Q0.75 = third quartile, Q0.25 = first quartile, IQR = difference between the third and first quartile).

The IQR outliers were marked on four variables: X coordinate, Y coordinate, speed, and distance. The average speed within the sliding window was also calculated. These labels will facilitate decision making when visually examining the GPS points and identifying noise points.

- 1. Identifying noise points in ArcGIS

We imported the GPS file into ArcGIS Pro after outlier labeling. Based on the instant speed and the sliding-window average speed (speed mean), the follow filters were applied to select potential noise GPS points. All confirmed noises were removed from the GPS dataset.

Filter A: speed outlier = true AND speed mean ≤ 0.5 m/s AND speed ≥ 4.35 m/s.

With an average speed lower than 0.5m/s but the instant speed over 4.35 m/s, the participant was likely in a sedentary status but the selected point had an abnormal high speed. If the selected GPS point appeared to be an outlier among a series of indoor points by checking against a base map, the point was marked as a noise. The threshold of 4.35 m/s was determined based on the world records of men’s race walking in 2015 (20km in 1 hour 16 minutes and 36 seconds, i.e. 4.35 m/s (https://athleticsweekly.com/stats/records/world-records-best-performances-mens-race-walking/)

Filter B: speed outlier = true and speed mean >0.5 and speed mean ≤ 4.35 and speed ≥4.35

With an average speed between 0.5 m/s and 4.35 m/s but the instant speed over 4.35 m/s, the participant was likely in a walking mode but the selected point had an abnormal high speed. If the selected GPS point appeared to be an outlier among a series of indoor points or points of walking mode in visual screening, the point was marked as a noise.

Filter C: speed outlier = true and speed mean >4.35 and speed mean ≤ 10.44 and speed ≥10.44

With an average speed between 4.35 m/s and 10.44 m/s but the instant speed over 10.44 m/s, the participant was likely in a running mode but the selected point had an abnormal high speed. If the selected GPS point appeared to be an outlier among a series of indoor points or points of running mode, the point was marked as a noise. The threshold of 10/44 m/s was determined based on the world record of100-meter sprint set by Usain Bolt in 2009 (https://www.britannica.com/story/how-fast-is-the-worlds-fastest-human).

Filter D: speed ≥ 36 (80.5 mph)

With an instant speed over 36 m/s but the point was not on a freeway, the participant was at an unrealistic speed so the selected GPS point was marked as a noise.

Filter E: speed outlier = true and distance outlier = true and (X outlier = true or Y outlier = true)

Lastly, other outliers previously labeled or not caught by the selections above during visual detection should also be marked as noises. Besides speed and spatial distribution, researchers also check the timestamps to facilitate their decision making.

1. Imputation of missing GPS

Due to loss of signals or removal of noises, we imputed the GPS to address the missing data issue. The imputation algorithm was derived from an existing study (Chambers et al., 2017). Missing GPS was imputed if one of the two criteria was met:

Condition A: known bookend coordinates for a gap must be less than 100 m apart and contiguous data gaps must be < 5 min.

Condition B: the distance between bookends was >100 m and the time gap was < 1 min.

A bookend was the point right before or after the gap of missing GPS data. The coordinates for missing intervals were assigned as the coordinates of the bookend prior to the data gap.
